# In-home assessment of flea control and dermatologic lesions in dogs provided by lotilaner (Credelio^®^) and spinosad (Comfortis^®^) in west central Florida

**DOI:** 10.1016/j.vpoa.2019.100009

**Published:** 2019-04-04

**Authors:** Michael W. Dryden, Michael S. Canfield, Brian H. Herrin, Cara Bocon, Todd S. Bress, Anna Hickert, Todd M. Kollasch, Letitia Phan, Anthony J. Rumschlag, William G. Ryan, Bridgette Sampeck, Nicole Smith, Vicki Smith, Stanislaw A. Warcholek

**Affiliations:** a Department of Diagnostic Medicine/Pathobiology, Kansas State University, Manhattan, KS 66506, USA; b Animal Dermatology South, 7741 Congress Str, New Port Richey, FL 34653, USA; c Elanco Animal Health, 2500 Innovation Way, Greenfield, IN 46140, USA; d Ryan Mitchell Associates LLC, 16 Stoneleigh Park, Westfield, NJ 07090, USA

**Keywords:** CADESI, canine atopic dermatitis extent and severity index, CPSS, canine pruritus severity scale, FAD, flea allergy dermatitis, GABA, γ-aminobutyric acid, KSU, Kansas State University, MB, mean baseline flea count prior to dosing, MA, mean flea count at subsequent visits, SD, standard deviation, Lotilaner, Spinosad, Dog, Flea, Field study, Dermatitis, Isoxazoline

## Abstract

Post-launch field investigations of recently-approved flea control products establish an efficacy baseline and in subsequent years can detect any efficacy decline suggestive of emerging resistance. As part of a continuing program of yearly assessment of flea control products in west central Florida, this study, using client-owned dogs, investigated the efficacy of lotilaner and spinosad in controlling fleas and in alleviating dermatologic signs likely associated with flea infestations. Forty-four qualifying households were randomized to either a lotilaner (Credelio^®^) (minimum dose rate 20 mg/kg) or a spinosad (Comfortis^®^) (30 mg/kg) group, with 33 and 36 dogs in each group, respectively. On Days 0 and 28 (±2) all dogs in each household were treated with the allocated product according to label directions, and all household cats received spinetoram (Cheristin^®^). On Day 0 and at weekly intervals through Day 56 (±2), on-animal and premises flea burdens were enumerated, a veterinary dermatologist scored integumental changes using canine atopic dermatitis extent and severity index (CADESI)-4 and flea allergy dermatitis (FAD) scales, and owners scored pruritus using the validated canine pruritus severity scale (CPSS).

At study entry geometric mean flea counts were 33.2 and 29.9 in the lotilaner and spinosad groups, respectively. For both groups, reductions in flea counts were > 99% at the first post-treatment assessment (Week 1), and 100% from Week 6 through the final assessment (Week 8) when all study dogs were flea-free. For both groups, at each timepoint, flea counts on dogs and in traps were significantly reduced compared to the initial assessment (p < 0.001), as were improvements in median CADESI-4, FAD and CPSS scores (p ≤ 0.001). At Week 4, the geometric mean flea count on dogs in the lotilaner group (0.1) was significantly lower than that of dogs in the spinosad group (0.6) (p = 0.027), significantly fewer dogs in the lotilaner group were found to have fleas (p = 0.034), and mean owner-rated pruritus scores were significantly lower (p = 0.025).

Under field conditions favoring heavy flea challenge, two consecutive monthly treatments of dogs with either lotilaner or spinosad produced a 100% reduction in canine flea infestations and dramatic improvements in dermatologic lesions and pruritus, based on scoring by a veterinary dermatologist and by dog owners. Household flea burdens were driven to extinction in all but one home in each treatment group.

## Highlights

• Field studies are important to monitor treatments against flea infestations.
• 2 monthly treatments with lotilaner were highly effective under severe challenge.
• 2 monthly treatments with spinosad quickly eliminated all fleas from dogs.
• Both treatments drove in-home flea biomass to extinction in all but one home.
• Both treatments led to marked improvement in dermatologic lesions and pruritus.

## 1 Introduction

The humid, subtropical climate of the Tampa Bay region on the west coast of Florida provides an ideal environment for the proliferation of flea burdens on household pets. These conditions offer an excellent opportunity to monitor the performance of flea control treatments under severe flea infestation pressure. Over the last three decades, the Clinical Parasitology Group in the Department of Diagnostic Medicine/Pathobiology at Kansas State University (KSU) has undertaken regular investigations in this region of many of the leading veterinarian recommended on-animal and oral flea control products. While immediate post-launch studies on flea control products are important to establish a baseline, studies in subsequent years can determine if efficacy continues to match the baseline and detect any loss of efficacy that could be an indicator of emerging resistance.

The isoxazolines are the most recent innovation to be approved for flea and tick control. The field studies required for USA registration of the first three isoxazolines—afoxolaner, fluralaner and sarolaner—compared these products with spinosad (Freedom of Information Summary, NexGard, 2013; Meadows et al., 2014; Cherni et al., 2016). Spinosad was introduced in 2007 as a highly effective orally-administered treatment for canine flea control and pioneered the move to monthly, systemic adulticide treatments for control programs. Early field studies with spinosad in the USA and Europe demonstrated excellent efficacy against existing flea infestations (Robertson-Plouch et al., 2008, Wolken et al., 2012, Dryden et al., 2013). Those studies and the recent isoxazoline-spinosad investigations have made spinosad one of the most field-tested flea control products, and the standard against which systemically active products are assessed.

To date, studies in west central Florida have investigated the efficacy of afoxolaner, fluralaner and sarolaner (Dryden et al., 2015, 2016, 2017, 2018). Herein we report a field investigation of the fourth-approved isoxazoline, lotilaner, to control flea populations and improve associated dermatologic lesions on dogs. Spinosad was selected as the reference control product because it is the only orally-administered, month-long, non-isoxazoline flea adulticide that has previously demonstrated its effectiveness in this area of Florida. This study also serves as a means of monitoring the ongoing efficacy of spinosad in West Central Florida USA.

## 2 Materials and methods

### 2.1 Households and animals

Referrals from Animal Dermatology South, New Port Richey, FL, and advertisements on Facebook allowed identification of 44 private residences that qualified for inclusion in the study. Household enrollment was initiated on May 15, 2018 and the final assessment of the last enrolled house was on July 20, 2018. Each qualifying home had at least one healthy, non-fractious dog, with no conditions that could affect study results. Homes with up to four additional healthy dogs or cats could qualify for the study (maximum of five household dogs and cats, combined). All enrolled pets were required to spend at least 12 h per day indoors. To meet label requirements for spinosad, dogs in enrolled homes had to be at least 14 weeks of age and weigh at least 2 kg. To ensure a valid challenge, at least five fleas had to be observed, using area flea counts, on at least one household dog, and at least five fleas collected in two intermittent-light flea traps left overnight at a pre-enrollment visit. Owners had to agree not to use any other topical or premise flea control products and to not bring any other dogs or cats into the household for the duration of the study. With the exceptions of maintaining a stabilized dose rate of any medication used to manage chronic conditions and administration of scheduled prophylactic treatments (*e.g*., heartworm preventives), no other veterinary drugs or treatments were to be given to any dog during the post-treatment period of the study.

### 2.2 Randomization and treatments

Each home that met all enrollment criteria was assigned a random number using Excel (Excel 2016, Microsoft, Redmond, WA) and blocked into groups of two. The higher random number within each block was assigned to group 1 and the lower to group 2. Group 1 dogs were treated orally with a lotilaner chewable tablet (Credelio^®^, Elanco Animal Health Incorporated) at a minimum dose rate of 20 mg/kg. Group 2 dogs were treated orally with a spinosad tablet (Comfortis^®^, Elanco Animal Health Incorporated) at a minimum dose rate of 30 mg/kg. Dogs had food available ad libitum or had been fed before or soon after dosing. All dogs and cats were weighed prior to each treatment to ensure proper dosing. Treatments were administered according to label directions on two occasions, once on Day 0, and once on Days 28–30. Dosing was completed by the veterinary student staff or by pet owners under staff observation. Cats in all enrolled households were treated topically with an 11.2% spinetoram spot-on solution (Cheristin^®^) administered according to label directions.

In some enrolled homes, not all dogs qualified for inclusion in the study (e.g., if one of the dogs had fewer than five fleas or was too fractious to examine). However, all qualifying and non-qualifying animals in each home were administered group-appropriate treatments.

### 2.3 Flea efficacy assessments

The flea population on each dog was assessed using a validated visual area count of five anatomic locations on each dog - the dorsal midline, the tail head, left lateral, right lateral, and inguinal region (Dryden et al., 1994). Counting at any location stopped at 50 fleas, so that the maximum flea count at any assessment was 250. The numbers of adult fleas emerging in the home was assessed using intermittent-light traps with adhesive pads to retain any fleas (Dryden and Broce, 1993, Müller et al., 2011). Two traps, one in each of two rooms, were left in place overnight for approximately 16–24 h. Room selection was based on where the dog(s) spent most of their time or where owners had observed fleas or where study staff observed large amounts of flea dirt, eggs or larvae. After collection of the traps the adhesive pads were removed and forwarded to the laboratory at KSU for counting and speciation of fleas under microscopic observation. The traps were returned to the same location in the same rooms at every counting period. Animal and premise flea counts were conducted on Day 0 and then at weekly intervals (±2 days) for 8 weeks.

### 2.4 Dermatology assessments

A board-certified veterinary dermatologist completed blinded clinical examinations of each study dog on Day 0 using the canine atopic dermatitis extent and severity index (CADESI)-4 and a flea allergy dermatitis (FAD) assessment tool (Wilkerson et al., 2004, Olivry et al., 2014, Dryden et al., 2016). At the time of enrollment and at each subsequent home visit a “primary” owner scored their dog’s level of “itchiness”, using a validated visual analog scale, the canine pruritus severity scale (CPSS) (Hill et al., 2007). On the CPSS, the owner would place a mark beside a description of scratching behavior they felt most appropriate for their dog. To determine the pruritus score, after each visit was concluded, in the absence of the owner, the location of the mark was assessed by matching it against a scale of 0 (itching not a problem) to 10 (extremely severe itching). With one exception the same primary owner assessed the pruritus level at each visit while remaining blinded to previous scores.

### 2.5 Safety assessments

All study dogs that received at least one treatment were monitored for adverse events. Any event observed by study staff or reported by owners was recorded in the study file. All adverse events were reported to the sponsoring company which then informed the relevant regulatory authority.

### 2.6 Data analysis

Efficacy within each treatment group at each assessment was defined as the percent reduction from baseline in geometric mean live adult on-dog flea counts and household intermittent-light flea trap counts. For flea count data in each group, descriptive statistics (number of cases, geometric mean, arithmetic mean, standard deviation, minimum, median, and maximum values) were presented for collections on Day 0 and Weeks 1 through 8. The percent change from Day 0 was calculated based on the geometric mean and the arithmetic mean for each group at Weeks 1 through 8. For geometric mean calculations, a natural log transformation was applied to flea/house count plus 1, and 1 was subtracted from the resulting mean value prior to calculation of efficacy. Percentage of control achieved by each product was calculated using the formula:

Percent efficacy = ([MB_0_ –MA]/ MB_0_)*100, where MB_0_ = mean baseline flea count prior to dosing (Day 0) and MA = mean flea count at subsequent visits (Days 28 and 56, ± 2 in each case).

The SAS Proc Mixed Procedure (SAS 9.3, Cary NC) was used for the statistical analysis. Possible differences between groups were assessed by repeated measures analysis of variance modeling of the natural log transformed counts with group, week and group by week interaction as fixed effects. Comparisons between groups at each week were generated from this model using linear contrast statements. The Wilcoxon signed rank test was used to evaluate within group comparisons from Day 0 to each week. The number and percentage of cases with zero flea counts were tabulated for each week and group. Possible differences between groups were assessed by the chi-square test.

For dermatology assessments in each group, descriptive statistics (number of cases, geometric and arithmetic means, standard deviation, minimum, median, and maximum values) were presented for scores collected on Day 0 and for subsequent study weeks. Possible differences between groups were assessed by repeated measures analysis of covariance modeling of the ranked values. The Day 0 value was included as a covariate with group, week and group by week interaction as fixed effects. Comparisons of median scores between groups at each assessment were generated from this model using linear contrast statements. The Wilcoxon signed rank test was used to evaluate within group comparisons from Day 0 to each week.

## 3 Results

### 3.1 Animals

There were 44 qualifying households enrolled into the study, 22 for each group. Within these households were 33 and 36 dogs qualifying for the lotilaner and spinosad groups, respectively. Overall group demographics were well balanced, except for the ratio of female to male which was 1.06 and 0.385 in the lotilaner and spinosad groups, respectively. The average age for dogs in each group was 6.5 years, with a similar range in each group (4 months to 15 years). Average weight in lotilaner-treated dogs was 17.7 kg (range 2.4–54.6) and in spinosad-treated dogs was 19.0 kg (2.3–58.2). The proportion of households with multiple dogs was 63.6 and 72.6% for the lotilaner and spinosad groups, respectively. There were five households in the lotilaner group that also had cats, and six in the spinosad group. Five dogs (7.2% of the total) in the study were reported to be receiving heartworm prophylaxis. There were 25 different dog breeds and cross breeds represented, with the most common breed descriptions being Pit Bull (including Pit Bull cross) (15) and Chihuahua (including Chihuahua cross) (14).

Three dogs in the lotilaner group, all from separate households, were lost to follow up, one after Week 2, one after Week 3 and one after Week 4. In the spinosad group eight dogs were lost to follow up, one after the Week 2 visit, three dogs from one household after Week 3, two from separate households after Week 4, and two from a single household after Week 6. In all cases, the reasons for dogs being lost to follow up were due to protocol violations, including owners moving or being unavailable, and none were attributed to adverse events or to treatment failure. All data recorded from the non-completing dogs were included in the analyses up to the point dogs were lost from the study. Occasional data points were missing from dogs in each group because of owner failure to make or keep appointments and neither the owner nor the dog(s) were available. Within groups there was some between-week variation in the numbers of dogs with flea counts, CPSS scores and dermatology scores. These variations arose when the dog was available for evaluation, but the primary owner was not present.

### 3.2 Flea control findings

At study entry geometric mean dog flea counts were similar between the two groups (lotilaner 33.2; spinosad 29.9) (Table 1). There were two dogs in the spinosad group with at least 250 fleas (the maximum count possible), while the maximum count in the lotilaner group was 175. In both groups, flea count reductions from baseline were > 99% at the first post-treatment assessment (one week after treatment) and were reduced by 100% from Week 6 through the final assessment on Week 8, when all study dogs were free of fleas. In the spinosad group, the maximum flea counts of 20, 36 and 14 for Weeks 2, 3 and 4 were all from a single dog, a 15-year-old Chihuahua-terrier cross that had a count of 91 fleas at enrollment and 8 fleas at Week 1. No fleas were observed on this dog at any assessment following the second treatment.

**Table 1:** On-animal flea counts in naturally infested dogs administered two monthly oral treatments of lotilaner or spinosad.

Study week	Group	n	Geometric Mean	Percent change from Day 0	Arithmetic mean	Percent change from Day 0	SD	Minimum	Median	Maximum	p-values*
											Between group
0	Spinosad	36	29.9	0.0	53.1	0.0	62.4	5.0	23.5	250.0	0.268
	Lotilaner	33	33.2	0.0	49.0	0.0	43.4	5.0	35.0	175.0	
1	Spinosad	36	0.2	99.4	0.5	99.1	1.7	0.0	0.0	8.0	0.312
	Lotilaner	33	0.3	99.1	0.4	99.1	0.7	0.0	0.0	3.0	
2	Spinosad	29	0.4	98.7	1.1	97.9	3.8	0.0	0.0	20.0	0.206
	Lotilaner	33	0.1	99.7	0.2	99.6	0.6	0.0	0.0	3.0	
3	Spinosad	34	0.4	98.5	1.5	97.1	6.2	0.0	0.0	36.0	0.337
	Lotilaner	32	0.2	99.4	0.3	99.3	0.8	0.0	0.0	3.0	
4	Spinosad	32	0.6	98.0	1.5	97.1	3.3	0.0	0.0	14.0	0.027
	Lotilaner	31	0.1	99.6	0.3	99.3	1.2	0.0	0.0	6.0	
5	Spinosad	30	0.1	99.8	0.1	99.8	0.3	0.0	0.0	1.0	0.515
	Lotilaner	26	0.2	99.5	0.3	99.5	0.7	0.0	0.0	3.0	
6	Spinosad	30	0.0	100.0	0.0	100.0	0.0	0.0	0.0	0.0	0.917
	Lotilaner	30	0.0	100.0	0.0	100.0	0.0	0.0	0.0	0.0	
7	Spinosad	28	0.0	100.0	0.0	100.0	0.0	0.0	0.0	0.0	0.985
	Lotilaner	30	0.0	100.0	0.0	100.0	0.0	0.0	0.0	0.0	
8	Spinosad	28	0.0	100.0	0.0	100.0	0.0	0.0	0.0	0.0	0.985
	Lotilaner	30	0.0	100.0	0.0	100.0	0.0	0.0	0.0	0.0	

SD Standard deviation.

* Within group p-values comparison to Day 0 generated by the Wilcoxon signed rank test. Week 1 through Week 8, for both groups p < 0.001. Between group p-values generated by repeated measures analysis of variance with Group, Week and Group by Week as fixed effects.

For both groups at all assessments, compared to study entry, the reductions in geometric mean flea counts were significant (p < 0.001). One week after the first treatment there was a significantly greater percentage of dogs on which no fleas were found in the spinosad group (88.9%) than in the lotilaner group (66.7%) (p = 0.025) (Table 2). At Week 4 when the geometric mean flea count reductions in the lotilaner and spinosad groups were 99.6 and 98.0%, respectively, the between-group geometric means were significantly different (p = 0.027) (Table 1). At this assessment there was also a significantly greater proportion of flea-free dogs in the lotilaner group (90.3%), relative to the spinosad group (68.8%) (p = 0.034).

**Table 2:** Number (percent) of dogs and households with zero flea counts.

Study week	Group	Dogs		Households	
		n (%)	p-value*	n (%)	p-value*
1	Spinosad	32 (88.9%)	0.025	4 (18.2%)	0.680
	Lotilaner	22 (66.7%)		3 (13.6%)	
2	Spinosad	21 (72.4%)	0.124	3 (15.8%)	0.839
	Lotilaner	29 (87.9%)		4 (18.2%)	
3	Spinosad	23 (67.6%)	0.207	6 (28.6%)	1.000
	Lotilaner	26 (81.3%)		6 (28.6%)	
4	Spinosad	22 (68.8%)	0.034	10 (50.0%)	0.197
	Lotilaner	28 (90.3%)		6 (30.0%)	
5	Spinosad	27 (90.0%)	0.543	14 (73.7%)	0.920
	Lotilaner	22 (84.6%)		13 (72.2%)	
6	Spinosad	30 (100.0%)	1.000	15 (78.9%)	0.935
	Lotilaner	30 (100.0%)		16 (80.0%)	
7	Spinosad	28 (100.0%)	1.000	16 (88.9%)	0.486
	Lotilaner	30 (100.0%)		19 (95.0%)	
8	Spinosad	28 (100.0%)	1.000	17 (94.4%)	0.939
	Lotilaner	30 (100.0%)		19 (95.0%)	

* Between group p-values generated by the Chi-square test.

For intermittent light trap results, at study entry geometric mean flea counts were 22.2 and 19.2 in the spinosad and lotilaner groups, respectively (Table 3). At all post-Day 0 assessments the reductions in geometric mean flea counts from traps were significant (p < 0.001) for both groups. The reduction from study entry in trap mean counts in both groups was >75% as soon as one week after treatment, and at least 80% from Week 4 until the end of the study when counts were zero in all but one home in each group. In each of those homes just two fleas were caught in the traps. There were no significant between-group differences in the percent of households in which flea trap counts were zero (p ≥ 0.197) (Table 2).

**Table 3:** Fleas recovered in premises traps in homes when dogs were administered two monthly oral treatments of lotilaner or spinosad.

Study week		n	Geometric mean	Percent change from Day 0	Arithmetic mean	Percent change from Day 0	SD	Minimum	Median	Maximum	p-values*
											Between group
0	Spinosad	22	22.2	0.0	46.4	0.0	107.8	5.0	23.0	524.0	0.642
	Lotilaner	22	19.2	0.0	30.8	0.0	36.4	4.0	20.0	152.0	
1	Spinosad	22	3.6	83.7	83.0	−78.8	366.2	0.0	2.0	1722.0	0.806
	Lotilaner	22	4.1	78.5	7.2	76.5	8.4	0.0	4.0	26.0	
2	Spinosad	19	3.8	82.8	64.4	−38.8	259.6	0.0	2.0	1136.0	0.895
	Lotilaner	22	3.9	79.4	8.9	71.1	15.5	0.0	3.0	70.0	
3	Spinosad	21	5.0	77.4	31.0	33.3	71.8	0.0	3.0	284.0	0.182
	Lotilaner	21	2.7	85.7	4.6	85.1	4.4	0.0	4.0	13.0	
4	Spinosad	20	1.7	92.5	10.2	78.1	33.0	0.0	0.5	148.0	0.815
	Lotilaner	20	2.1	89.0	4.1	86.8	6.2	0.0	2.0	26.0	
5	Spinosad	19	0.5	97.6	1.9	95.9	5.5	0.0	0.0	23.0	0.368
	Lotilaner	18	0.5	97.5	1.1	96.6	2.5	0.0	0.0	10.0	
6	Spinosad	19	0.3	98.5	0.8	98.3	2.0	0.0	0.0	7.0	0.316
	Lotilaner	20	0.2	98.8	0.5	98.5	1.4	0.0	0.0	6.0	
7	Spinosad	18	0.2	99.3	0.4	99.2	1.4	0.0	0.0	6.0	0.169
	Lotilaner	20	0.1	99.7	0.1	99.7	0.5	0.0	0.0	2.0	
8	Spinosad	18	0.1	99.7	0.1	99.8	0.5	0.0	0.0	2.0	0.257
	Lotilaner	20	0.1	99.7	0.1	99.7	0.5	0.0	0.0	2.0	

SD Standard deviation.

* Within group p-values (based on geometric means) comparison to Day 0 generated by the Wilcoxon signed rank test. Week 1 through Week 8, for both groups p < 0.001. Between group p-values generated by repeated measures analysis of variance with Group, Week and Group by Week as fixed effects.

At the enrollment visit for one household five fleas were initially counted in the retrieved traps. However, under microscopic examination at KSU one of the counted fleas was found to be a wingless fly (scuttle fly; Puliciphora) with a general shape and size very similar to a flea. Although not meeting the entry criterion of a trap count of five fleas, the household was retained in the study because the live flea counts of 78 and 19 on the two dogs that were present was considered adequate to ensure that there was a substantial flea challenge at study initiation. Other than this one instance of initial incorrect identification, all 6099 fleas collected in the traps throughout the study were identified as *Ctenocephalides felis felis.*

During the study three households met the description of a red-line home (an increase in premises trap counts of at least 20% over day 0 within 1–4 weeks post-treatment) (Dryden, 2009, Dryden et al., 2011). In one of these, a single-dog household randomized to the spinosad group, trap counts increased from 524 at baseline to 1722 at Week 1, declined to 1136 at Week 2, 284 at Week 3 and 26 at Week 4. No further data were available because the owner moved into an assisted-care living facility, forcing the withdrawal of the dog from the study. The enrollment flea count on the dog was 250 (the maximum before counting stopped), declining to 2 at Week 1 and 0 at Week 4. Additionally, this dog showed improvement in all dermatology variables following the spinosad treatment.

### 3.3 Dermatology assessments

Median CADESI-4 scores at enrollment were 21.0 and 19.5 in the lotilaner and spinosad groups, respectively (Table 4). Benchmarks proposed for mild, moderate and severe atopic dermatitis skin lesions are 10, 35 and 60, respectively, so that a normal dog score is <10, a mild atopic dermatitis score is from 10 to 34, moderate from 35 to 59 and severe ≥60 (Olivry et al., 2014). On this basis, at enrollment 80.7% of lotilaner-treated dogs and 78.1% of spinosad-treated dogs had CADESI-4 scores that would be considered mild to severe in dogs with atopic dermatitis, with three dogs in each group being severely affected. At the end of the study median and mean CADESI-4 scores in each group had declined to be within a normal dog range, only one dog in each group was scored as moderately affected, and no dog scored as severe (Table 5). Between the enrollment and subsequent assessments there was a significant reduction in median scores for both groups in CADESI-4 (p ≤ 0.001) and FAD scores (p ≤ 0.001), with no significant differences between groups at any time (p ≥ 0.236).

**Table 4:** Assessment of skin lesions using a canine atopic dermatitis extent and severity index (CADESI)-4 score and flea allergy dermatitis score for dogs naturally infested with fleas and administered two monthly oral treatments of lotilaner or spinosad.

Study week	Group	n						p-values*	
			Median	Mean	SD	Minimum	Maximum	Within group	Between group
Canine atopic dermatitis and severity index (CADESI)-4 score									
0	Spinosad	32	19.5	25.7	19.5	4.0	65.0		0.799
	Lotilaner	31	21.0	24.4	19.0	3.0	80.0		
4	Spinosad	32	13.5	18.3	16.5	1.0	57.0	0.001	0.236
	Lotilaner	31	10.0	14.4	13.1	1.0	56.0	<0.001	
8	Spinosad	28	6.0	8.1	9.1	0.0	36.0	<0.001	0.794
	Lotilaner	30	4.0	7.4	10.1	0.0	52.0	<0.001	

Flea allergy dermatitis scores									
0	Spinosad	32	9.0	12.1	10.1	0.0	37.0		0.242
	Lotilaner	31	9.0	9.4	7.7	0.0	30.0		
4	Spinosad	32	5.5	8.1	7.7	1.0	26.0	0.002	0.321
	Lotilaner	31	3.0	4.2	4.0	0.0	14.0	<0.001	
8	Spinosad	28	1.0	3.3	5.0	0.0	18.0	<0.001	0.343
	Lotilaner	30	1.0	1.4	1.9	0.0	7.0	<0.001	

ANCOVA was generated based on ranked values.

SD Standard deviation.

* Within group p-values comparison to Day 0 generated by the Wilcoxon signed rank test. Between group p-values generated by repeated measures analysis of covariance with Day 0 as a covariate and Group, Week and Group by Week as fixed effects.

**Table 5:** Number (percent) of dogs with each canine atopic dermatitis extent and severity index (CADESI)-4 classification before and after administration of two monthly oral treatments of lotilaner or spinosad.

Study week	Group	Normal	Mild	Moderate	Severe
0	Spinosad	7 (21.9)	16 (50.0)	6 (18.8)	3 (9.3)
	Lotilaner	6 (19.4)	20 (64.5)	2 (6.5)	3 (9.7)
4	Spinosad	12 (37.5)	14 (43.8)	6 (18.8)	0 (0.0)
	Lotilaner	14 (45.2)	14 (45.2)	3 (9.7)	0 (0.0)
8	Spinosad	20 (71.4)	7 (25.0)	1 (3.6)	0 (0.0)
	Lotilaner	25 (83.3)	4 (13.3)	1 (3.3)	0 (0.0)

In each group median FAD assessment scores at study entry were 9.0, declining to 1.0 at Week 8 (Table 4). Scores for all but two dogs completing the study declined between Week 0 and Week 8. Those two dogs had low starting FAD assessment scores of 0 and 2.

Using the owner-assessed CPSS, the range of 0–1.9 is regarded as being indicative of a normal dog (Rybnícek et al., 2009). At study entry CPSS scores for all but three dogs exceeded 1.9 (Table 6). Median scores, considered of greater value than means for determining a response to treatment, were ≤1.9 in the lotilaner group for Weeks 4 through 8 and in the spinosad group from Weeks 5 through 8 (Rybnícek et al., 2009). At Week 4, the median CPSS scores of 1.7 for lotilaner and 4.5 for spinosad were significantly different (p = 0.025).

**Table 6:** Owner-rated canine pruritus severity scale scores, descriptive statistics and p-values.

			Dogs with score ≤1.9						p-values*
	Group	n	n (%)	Median	Mean	SD	Minimum	Maximum	Between group
Day 0	Spinosad	36	3 (8.3%)	7.4	6.5	2.7	1.4	10.0	0.152
	Lotilaner	33	0 (0.0%)	7.4	7.3	1.7	3.5	10.0	
Week 1	Spinosad	36	8 (22.2%)	3.5	4.1	2.3	0.0	8.5	0.971
	Lotilaner	33	9 (27.3%)	3.3	3.4	2.0	0.0	7.4	
Week 2	Spinosad	28	8 (28.6%)	3.5	3.3	2.1	0.0	9.0	0.896
	Lotilaner	33	15 (45.5%)	2.7	3.0	2.2	0.0	7.5	
Week 3	Spinosad	31	8 (25.8%)	3.5	3.5	2.1	0.0	7.6	0.274
	Lotilaner	32	13 (40.6%)	2.0	2.5	2.2	0.0	7.4	
Week 4	Spinosad	32	8 (25.0%)	4.5	4.1	2.4	0.0	9.0	0.025
	Lotilaner	31	17 (54.8%)	1.7	2.2	1.8	0.0	6.0	
Week 5	Spinosad	30	15 (50.0%)	1.9	2.4	1.9	0.0	8.9	0.720
	Lotilaner	26	20 (76.9%)	1.5	1.7	1.7	0.0	5.7	
Week 6	Spinosad	30	22 (73.3%)	1.4	2.0	2.2	0.0	7.6	0.973
	Lotilaner	30	23 (76.7%)	1.2	1.3	1.3	0.0	6.4	
Week 7	Spinosad	26	25 (96.2%)	0.7	1.0	1.1	0.0	5.5	0.084
	Lotilaner	30	25 (83.3%)	0.6	1.2	1.6	0.0	6.2	
Week 8	Spinosad	28	20 (71.4%)	1.0	1.4	1.6	0.0	5.9	0.930
	Lotilaner	28	23 (82.1%)	0.5	1.1	1.3	0.0	5.2	

* Within group p-values comparison to Day 0 generated by the Wilcoxon signed rank test. Week 1 through Week 8, for both groups p < 0.001. Between group p-values generated by repeated measures analysis of covariance with Day 0 as a covariate and Group, Week and Group by Week as fixed effects. ANCOVA was generated based on ranked values. SD Standard deviation.

### 3.4 Safety assessments

There were three reported adverse events in the lotilaner group, none of which were attributed to treatment, although the role of treatment as a cause of transient anorexia reported for two dogs in a single home one to two days post-treatment could not be entirely discounted. In another household, diarrhea was reported by the owner to have occurred eight hours after the dog was treated. A fecal test identified infection with the hookworm *Ancylostoma caninum* as the cause of the diarrhea, and the dog recovered following treatment with a combination product containing praziquantel, pyrantel pamoate and febantel (Drontal^®^ Plus, Bayer).

In the spinosad group there were six reports of gastrointestinal events, one in each of six dogs. In one household each of two dogs vomited within two hours following the first treatment, and the owner also reported an observation of “inappropriate defecation” by the dogs. The dogs were not re-dosed until the next scheduled treatment. No fleas were found on either dog at any post-treatment assessment and there was no vomiting or other adverse event following the second scheduled treatments. In a second household two dogs vomited two hours following their second monthly treatment and were not redosed. Flea counts were zero on those dogs in the two weeks following this treatment, after which this home was non-responsive to requests for appointments and was withdrawn from the study. In a third household, nine days after treatment the owner reported transient episodes of vomiting after the dog had been fed table scraps, while in a fourth household an enrolled dog vomited 24 h after treatment. All reported events were mild to moderate in degree and dogs recovered uneventfully without treatment.

## 4 Discussion

The on-animal flea count methodology used in this and previous in-home investigations conducted by these authors, detects approximately 23.5% of the total dog flea burden (Dryden et al., 1994). Therefore, based on geometric mean area counts of 33.2 and 29.9, the estimated Day 0 total flea burdens of dogs treated with lotilaner or spinosad can be estimated to be 141 (range 21–744) and 127 (range 21–1063), respectively. These numbers clearly indicate that the dogs enrolled in this study had extremely large natural flea infestations.

Lotilaner and spinosad both demonstrated rapid and sustained efficacy in controlling fleas under extreme natural flea challenge, including in red-line homes. The efficacy response to each product, with associated improvement in dermatology (including CPSS) scores, can be attributed to the rapid knockdown of fleas (residual speed of kill) that was sustained throughout each month following treatment. Laboratory studies with spinosad have demonstrated that 100% of fleas infesting dogs are killed within four hours post-treatment, with flea killing evident within one hour after treatment (Franc and Bouhsira, 2009, Blagburn et al., 2010). The onset of flea-killing activity has been demonstrated to be quicker for spinosad than for afoxolaner, based on assessments completed at 1 and 3 h post treatment, and at one hour following an infestation on Day 7 (Snyder et al., 2015). That speed of onset may have been a factor in our Week 1 finding of significantly more dogs being free of fleas in the spinosad group (88.9%) than in the lotilaner group (66.7%) (p = 0.025). The field efficacy of spinosad that we report aligns with findings over the years of other studies in the USA and Europe demonstrating that spinosad continues to be as effective now as when first made available in 2007 (Robertson-Plouch et al., 2008; Wolken et al., 2012; Dryden et al., 2013; Meadows et al., 2014; Freedom of Information Summary, NexGard, 2013; Hayes et al., 2015; Becskei et al., 2016; Cherni et al., 2016; Dryden et al., 2017). Nonetheless, the significant between-treatment differences at Week 4, both in owner scoring of the CPSS and in flea counts, merit further consideration, although these differences were not observed during the remainder of the study.

It is of interest that of the 69 dogs in the current study, 66 (95.7%) were scored by their owners on the CPSS as being abnormally pruritic at enrollment (i.e., having a pruritus score >1.9) (Rybnícek et al., 2009). In two previous studies conducted by the KSU flea team in west central Florida using this CPSS system, at study initiation 61 out of 61 (100%) and 51 of 53 (96.2%) dogs had owner-rated CPSS scores indicative of an abnormal level of pruritus (Dryden et al., 2015, Dryden et al., 2016). Therefore, based on this validated owner scoring methodology, of 183 dogs enrolled in these studies 178 (97.3%) were abnormally pruritic. The cumulative findings therefore demonstrate that the “area count” five-flea minimum enrollment criterion utilized in these studies is appropriate for the inclusion of dogs that are assessed by their owners as being abnormally pruritic.

From the many field reports describing comparisons of spinosad with other flea control products, our study is just the second to find a significant improvement over spinosad (Robertson-Plouch et al., 2008; Wolken et al., 2012; Dryden et al., 2013; Meadows et al., 2014; Freedom of Information Summary, NexGard, 2013; Hayes et al., 2015; Becskei et al., 2016; Cherni et al., 2016; Dryden et al., 2017). Meadows et al. (2014) found a significant difference in favor of fluralaner over spinosad in the proportion of flea-free dogs four weeks after each of the first and the third monthly spinosad treatments, but there was no evidence that this difference was clinically relevant. Our study is the first in which a significant efficacy improvement over spinosad has been demonstrated, and in which that improvement has been linked to a clinical finding. The significant differences in both studies may be due to the extended half-lives of fluralaner and lotilaner compared with spinosad. Following oral administration to recently fed dogs the half-life of spinosad is reported to be approximately five days, that of fluralaner approximately 14 days and of lotilaner approximately 30 days (European Medicines Agency. Comfortis: Summary of Product Characteristics, 2007; Kilp et al., 2014; Toutain et al., 2017).

For lotilaner, these results align with laboratory and earlier field studies. In a laboratory study, fleas were observed to become moribund on lotilaner-treated dogs as soon as one hour after treatment, and by two hours the geometric mean live flea count reduction was 64.0%, with many of the live fleas classified as moribund (Cavalleri et al., 2017a). The moribund state would likely render fleas unable to recover or feed and therefore not to be a cause of flea-bite related pruritus. In another laboratory study, lotilaner provided sustained rapid efficacy against post-treatment flea infestations, with 100% of fleas killed within 12 h (Cavalleri et al., 2017b). In a field study in the USA, based on geometric means, the efficacy of three consecutive monthly lotilaner treatments was 99.3, 99.9 and 100% on Days 30, 60 and 90, with 100% of lotilaner-treated dogs and 93% of afoxolaner-treated dogs flea-free at the final assessment (Karadzovska et al., 2017). Lotilaner results in a European field study were similar to those of the USA study, with significant differences in favor of lotilaner treatment relative to a control group treated with fipronil (Cavalleri et al., 2017c).

The ability of both products to rapidly and continuously kill on-dog fleas resulted in a progressive reduction to extinction or near-extinction of emergent fleas. Of interest were the on-dog and intermittent-light trap flea counts from one, specific spinosad-group household. At baseline, the dog had too many fleas to count (recorded as the maximum of 250) and trap counts identified 524 fleas. At Week 1, trap counts had increased to 1722, making this a true red-line home, but only two fleas were identified on the dog. At Week 2, trap counts had declined substantially, and by Week 4 (when the owner moved to an assisted-living care facility) the combined count from both traps was 26, at which time the dog was free of fleas. The findings of large-scale flea emergence early in the study, low post-treatment counts on the dog, and the decline in flea trap counts demonstrates the “vacuuming effect” of a monthly flea treatment with a product such as spinosad or lotilaner. Fleas that emerge and infest a treated dog are rapidly killed, before laying any eggs, so that there is no addition to household immature flea life-stage bio-mass. There is therefore a progressive depletion in environmental flea burden. Even though flea trap counts remained high due to a large preexisting immature flea life-stage biomass in the early post-treatment period, this dog showed a progressive improvement in all dermatology variables until being withdrawn because of the owner’s circumstances.

Both isoxazolines and spinosad have repeatedly demonstrated high sustained efficacy and residual insecticidal activity that are mediated through binding to different target sites (Kirst, 2010, Rufener et al., 2017). The results for lotilaner in this field study are consistent with the findings of earlier studies completed in this region of Florida investigating the field performance of the three other currently available isoxazolines. All were 99–100% effective from the first week after initiating treatment (Dryden et al., 2015, 2016, 2017, 2018). The sustained efficacy and residual speed of kill of these compounds, along with their efficacy against ticks and other acarines, are remarkable and signify a substantial breakthrough in the control of canine ectoparasite infestations.

Treatments were administered according to schedule to all dogs and cats in qualifying households in which observations were continued to Week 4 and beyond. Those treatments included non-study dogs and cats. Such treatment of all dogs and cats in a household is an important factor in minimizing household flea biomass and therefore in controlling flea challenge to household pets.

An unexpected finding from our routine medical history screening questionnaire was the very limited use of heartworm preventives in the enrolled canine population. Just five dogs (7.2% of the total) were receiving this prophylaxis despite being in an area in which heartworm infection is regarded as presenting significant risk to canine health. These failures in use of heartworm preventives are likely due to a combination of factors, including inadequate education of dog owners of the risks of their dog’s infection with *Dirofilaria immitis*, and the economic circumstances that place prophylaxis beyond the means of some dog owners. We feel it is incumbent on the pharmaceutical suppliers of heartworm preventives and practicing veterinarians to address this problem as a means of reducing the risk of heartworm infection of all dogs.

Both products were well tolerated, with a few observations of post-treatment vomiting by some spinosad-group dogs. This is a well-recorded effect of spinosad treatment that can occur in a small proportion of dogs and was not considered to present any safety risk. There was no reduction in efficacy observed in dogs that vomited at approximately two hours post treatment and were not re-dosed. For lotilaner, the study confirmed the safety that has been reported from early laboratory work demonstrating a wide safety margin, including in young puppies, and from field reports (Cavalleri et al., 2017c, Karadzovska et al., 2017, Kuntz and Kammanadiminti, 2017). The safety of lotilaner has been attributed to its exclusive selective binding to insect GABA-gated chloride channel receptors while not having the capacity to bind to mammalian receptors so that potent insecticidal potency is retained along with a wide safety margin (Rufener et al., 2017).

## 5 Conclusions

Under field conditions favoring heavy flea challenge in the subtropical climate of west central Florida, two consecutive monthly treatments of dogs with either lotilaner or spinosad were safe and produced a 100% reduction in canine flea infestations leading to marked improvement in dermatologic lesion scores and owner-scored pruritus. In the 38 enrolled households that completed the 2-month study, premises flea burdens were driven to extinction in all but one home in each treatment group.

## Conflict of interest

The studies reported here were funded by Elanco Animal Health, Greenfield, IN. AJR and TMK are employees of Elanco Animal Health. MWD has had research projects funded at Kansas State University and lectures sponsored by Elanco Animal Health, manufacturers of Credelio, Comfortis^®^ and Cheristin™ that were evaluated in this investigation. WGR has acted as a paid consultant for Elanco. MC has provided lectures sponsored by Elanco. BHH has had projects and lectures funded by Elanco. There were no conflicting interests that could have influenced the conduct and reporting of these studies.

## Funding

This study was funded by a grant from Elanco Animal Health Incorporated, Greenfield, IN, USA.

## Availability of data and materials

The datasets supporting the conclusions of this article are included within the article. Raw data from this study has not been shared at this time because we anticipate additional publications exploring some of the detailed and complex dermatologic results.

## Author contributions

MWD and BHH were primary authors of study design, served as primary study investigators and assisted in manuscript revision. WGR drafted the manuscript and assisted in study design and was the primary study monitor. VS coordinated and supervised data collection and entry. AH, BS, CB, LP, NS, SAW and TSB were responsible for animal handling, collection of data and data entry. MC was responsible for designing and completing blinded dermatology assessments and assisting in study design. AJR and TMK assisted in design of study, monitoring of study and manuscript revision. All authors reviewed and approved the final manuscript.

## Ethics approval and consent to participate

The protocol was reviewed and approved as complying with the regulations set forth by the Kansas State University Institutional Animal Care and Use Committee. This research protocol IACUC # 4086 was approved 10 May 2018. Each pet owner was required to receive a written and verbal explanation of the study protocol and the study products to be used, and was required to sign an informed consent form prior to enrolling their dog(s) into this field trial.

## Consent for publication

Not applicable.
